# Excess mortality in Wuhan city and other parts of China during the three months of the covid-19 outbreak: findings from nationwide mortality registries

**DOI:** 10.1136/bmj.n415

**Published:** 2021-02-24

**Authors:** Jiangmei Liu, Lan Zhang, Yaqiong Yan, Yuchang Zhou, Peng Yin, Jinlei Qi, Lijun Wang, Jingju Pan, Jinling You, Jing Yang, Zhenping Zhao, Wei Wang, Yunning Liu, Lin Lin, Jing Wu, Xinhua Li, Zhengming Chen, Maigeng Zhou

**Affiliations:** 1 The National Center for Chronic and Non-communicable Disease Control and Prevention, Chinese Center for Disease Control and Prevention (China CDC), Xicheng District, 100050, Beijing, China; 2 Hubei Provincial Center for Disease Control and Prevention, Wuhan, Hubei, China; 3 Wuhan Center for Disease Control and Prevention, Wuhan, Hubei, China; 4 Chinese Center for Disease Control and Prevention, Beijing, China; 5 Nuffield Department of Population Health, University of Oxford, Oxford, UK

## Abstract

**Objective:**

To assess excess all cause and cause specific mortality during the three months (1 January to 31 March 2020) of the coronavirus disease 2019 (covid-19) outbreak in Wuhan city and other parts of China.

**Design:**

Nationwide mortality registries.

**Setting:**

605 urban districts and rural counties in China’s nationally representative Disease Surveillance Point (DSP) system.

**Participants:**

More than 300 million people of all ages.

**Main outcome measures:**

Observed overall and weekly mortality rates from all cause and cause specific diseases for three months (1 January to 31 March 2020) of the covid-19 outbreak compared with the predicted (or mean rates for 2015-19) in different areas to yield rate ratio.

**Results:**

The DSP system recorded 580 819 deaths from January to March 2020. In Wuhan DSP districts (n=3), the observed total mortality rate was 56% (rate ratio 1.56, 95% confidence interval 1.33 to 1.87) higher than the predicted rate (1147 *v* 735 per 100 000), chiefly as a result of an eightfold increase in deaths from pneumonia (n=1682; 275 *v* 33 per 100 000; 8.32, 5.19 to 17.02), mainly covid-19 related, but a more modest increase in deaths from certain other diseases, including cardiovascular disease (n=2347; 408 *v* 316 per 100 000; 1.29, 1.05 to 1.65) and diabetes (n=262; 46 *v* 25 per 100 000; 1.83, 1.08 to 4.37). In Wuhan city (n=13 districts), 5954 additional (4573 pneumonia) deaths occurred in 2020 compared with 2019, with excess risks greater in central than in suburban districts (50% *v* 15%). In other parts of Hubei province (n=19 DSP areas), the observed mortality rates from pneumonia and chronic respiratory diseases were non-significantly 28% and 23% lower than the predicted rates, despite excess deaths from covid-19 related pneumonia. Outside Hubei (n=583 DSP areas), the observed total mortality rate was non-significantly lower than the predicted rate (675 *v* 715 per 100 000), with significantly lower death rates from pneumonia (0.53, 0.46 to 0.63), chronic respiratory diseases (0.82, 0.71 to 0.96), and road traffic incidents (0.77, 0.68 to 0.88).

**Conclusions:**

Except in Wuhan, no increase in overall mortality was found during the three months of the covid-19 outbreak in other parts of China. The lower death rates from certain non-covid-19 related diseases might be attributable to the associated behaviour changes during lockdown.

## Introduction

In China the emergence of a severe acute respiratory syndrome-like atypical pneumonia was first reported during mid-December 2019 in Wuhan city, Hubei province.^1^ ^2^ This led to identification in early January 2020 of a novel β coronavirus that the Word Health Organization subsequently designated as severe acute respiratory syndrome coronavirus 2 (SARS-CoV-2) and that causes an illness called coronavirus disease 2019 (covid-19).^3^ Coinciding with festivities for the Chinese lunar new year during January 2020,^4^ ^5^ however, SARS-CoV-2 spread rapidly to many other provinces in China and then worldwide within a short period.^4^ ^6^ ^7^ By early July 2020, more than 10 million people worldwide were reported to have been infected with SARS-CoV-2, among whom more than 500 000 were reported to have died from covid-19.^8^

In China, unprecedented nationwide measures were introduced and implemented from late January 2020 to contain, suppress, and eliminate the initial outbreak and further transmission of SARS-CoV-2. The nationwide lockdown, first implemented in Wuhan on 23 January 2020, coupled with other measures such as widespread testing, contact tracing, and quarantine of infected people at home and subsequently in purpose built temporary hospitals,^9^ ^10^ ^11^ successfully controlled the epidemic by the end of March. Consequently, the lockdown was lifted in all provinces of China from early or mid-April 2020, including Wuhan city.

By the end of March, more than 80 000 people had been infected in China (about 50 000 in Wuhan) and more than 4600 (about 3870 in Wuhan) deaths were recorded as being due to covid-19. Previous studies in China have reported on the molecular, clinical, and epidemiological characteristics of SARS-CoV-2 and covid-19,^4^ ^12^ ^13^ in addition to the effects of different preventive measures to contain and suppress the outbreak.^14^ ^15^ No study has, however, systematically examined overall and cause specific mortality across different parts of China during the covid-19 outbreak. Such evidence is needed to help assess the likely detrimental impact of the outbreak on human health and the healthcare system, and to inform future public health emergency responses and the provision of health services during major outbreaks.

We investigated the excess total and cause specific mortality during three months of the covid-19 outbreak (January-March 2020) across different regions of China, based on a nationally representative sample of more than 300 million people.^9^

## Methods

### Study population

We obtained information on our study population from China’s Disease Surveillance Points (DSP) system, which was established during the mid-1980s to provide nationally representative mortality statistics for China.^9^ ^16^ ^17^ The DSP system currently comprises 605 surveillance areas, involving more than 300 million people—more than 20% of the total population in China (see supplementary fig 1). Each surveillance area covers an urban district or a rural county, which was randomly selected using a multistage cluster sampling method across all 31 provinces (or equivalent) in mainland China to ensure representativeness at provincial and national levels. For each DSP, the population data (eg, number, age, and sex distributions) were extracted and updated annually from the National Bureau of Statistics.

### Mortality surveillance

In each DSP area, local Centers for Disease Control and Prevention (CDC) managed the death registry at district or county level. Qualified medical staff in local hospitals usually determined the causes of death and coded these according to ICD-10 (international classification of diseases, 10th revision) codes. For deaths that occurred outside of hospital without recent medical attention, trained staff at local community health centres determined the causes of death using standardised procedures.^9^ ^18^ To minimise any underreporting of deaths, regular checks and updates were conducted using other data sources (eg, police stations, the civil affairs department), supplemented by active surveys every three years involving a random sample (5-6%) of households in all catchment areas. Dedicated local hospital (for deaths in hospital) or health centre (for deaths outside of hospital) staff reported all deaths online through China CDC’s Death Information System. Before an online report was submitted to China CDC, the information in death certificates that had been uploaded online were reviewed and checked, and, if necessary, coded or recoded by local (district or county) CDC staff. Any deaths that occurred outside of DSP catchment areas would be linked back to the original address of residence. Once China CDC had received these reports it undertook additional checks to ensure completeness, consistency, and data quality, including the proportion of ill defined causes of death (typically <5%).

In Wuhan city, apart from the three DSP districts, the remaining 10 non-DSP districts also reported deaths using an identical online system from 1 January 2019. We also included these data in our analyses. Among the 13 districts, six were in central city and seven in suburban areas (see supplementary fig 1).

### Reporting of covid-19 deaths

As covid-19 is a newly emerged infectious disease without a formal ICD code in the online reporting systems, most early deaths from covid-19, whether confirmed or suspected, during the outbreak in Wuhan were typically notified as “viral pneumonia without known organism” (ICD-10 codes J12, J12.8, and J12.9) or “unspecific pneumonia” (ICD-10 code J18.9). From 2 February 2020, the China Health Commission requested that any pneumonia related deaths, including those from covid-19, were to be reported online to China CDC within five days through its DSP mortality surveillance reporting system and separately through its infectious disease surveillance system.^9^ For confirmed covid-19 deaths a new ICD-10 code (U07.1) was used, whereas for suspected covid-19 deaths (without microbiological confirmation), an existing code (J12.8) was used.

During April 2020 in Wuhan city, local health authorities conducted further systematic cross checks of death records from different sources (eg, hospitals, infectious disease reporting system, death registries, civil affairs departments, directors of funeral services), resulting in the identification of 1290 additional deaths from covid-19, including a proportion of deaths with a clinical diagnosis but no microbiological confirmation of covid-19.^13^ ^19^ ^20^ ^21^ Of these 1290 deaths, 89% were already captured in the DSP system and about 23% were reclassified as covid-19 from other causes. So, unlike other diseases, the reporting of deaths due to covid-19 and other pneumonias caused by other pathogens (ie, not SARS-CoV-2) or pneumonia probably due to SARS-CoV-2 but not initially recorded as such was likely to be complete by late May 2020.

### Statistical analyses

The main analyses involved deaths at any age during the first quarter of 2020 in DSP areas that were reported to China CDC by 22 May 2020. Supplementary table 1 shows the main diseases and corresponding ICD-10 codes. The deaths were classified into three broad categories: infectious diseases (also included were a small number of deaths from maternal, perinatal, and nutritional conditions), chronic non-communicable diseases, and injuries. Within each category, we also examined a limited number of individual diseases separately, including deaths due to different types of pneumonia (eg, unspecified viral pneumonia, covid-19 related pneumonia), cardiovascular disease (myocardial infarction, ischaemic stroke, haemorrhagic stroke, and hypertensive heart disease), or injury (eg, road traffic incident, suicide, and fall).

Analyses were conducted and presented separately for three main areas in DSP according to the severity of the covid-19 epidemic: Wuhan (n=3 DSP districts), Hubei province without Wuhan (n=19 DSP areas), and China without Hubei province (n=583 DSP areas). Apart from three DSP districts, 2019-20 data from the other 10 non-DSP districts in Wuhan were also available, which we examined and compared both overall and separately in central and suburban districts, in combination with the three DSP districts.

Based on the 2015-19 data, we estimated any possible delays in reporting of deaths for each week across different DSP areas in 2020 (see supplementary table 2). The weekly adjustment ratio was calculated as the mean weekly number of deaths occurring during January-March in 2015-19 that were reported by 22 May each year divided by the mean weekly number of deaths for the same period reported by the end of February the following year (when the database for the previous year was frozen). We adjusted the deaths in 2020 for probable delay in reporting (except for pneumonia related deaths), calculated by dividing the observed weekly mortality rates by the adjustment ratio for each province (see supplementary table 2).

Because the population data for 2020 are not currently available, we used the 2019 population in each DSP area to calculate weekly or quarterly mortality rates in 2020 (see supplementary table 3), which were then multiplied by 52 or 4, respectively, to yield annual mortality rates to facilitate comparisons. While estimating weekly mortality, we applied the same denominators over time (ie, assuming a constant weekly population), as the number of weekly deaths (≤40 000) was small compared with the size of the study population (>300 million). Based on the mortality rates calculated separately for 2015-19 and the five year trends, we estimated the predicted rates for 2020, overall and by time period and areas. In addition, we also calculated the mean mortality rates for 2015-19 to facilitate comparisons by, for example, age, sex, and place of death. We calculated these separately by individual DSP areas (at district or county level) and then summed accordingly with necessary weighting by population sizes.

For each disease, the weekly expected number of deaths from 1 January to 31 March 2020 were generated by modelling the observed weekly number of deaths occurring during 2015-19 (see supplementary fig 2) using Farrington surveillance algorithms.^22^ The Farrington algorithms, implemented by the surveillance package in R,^23^ used over-dispersed Poisson generalised linear models with spline terms to estimate trends in weekly counts of deaths that could account for seasonality, along with the lower and upper 95% confidence intervals of weekly predicted counts. For each disease, we calculated the weekly observed and predicted mortality rates along with corresponding 95% confidence intervals from the weekly observed and predicted number of deaths divided by the population in different DSP areas, respectively. To further enable comparison, the observed and predicted weekly mortality rates (and 95% confidence intervals) were summed from January to March in 2020 and then multiplied by 4 to yield annual mortality rates. To estimate the excess mortality during the study period, the rate ratios for mortality due to each disease were calculated by dividing the observed mortality rates by the predicted mortality rates, with each 95% confidence interval calculated similarly by the corresponding upper and lower bounds of mortality rates.

### Patient and public involvement

The mortality data of the study population were collected through the DSP system, which included age, sex, national identification number, residential address, cause of death and ICD-10 code, and date and place of death. The information used in this report is managed by China CDC and is not accessed by patients and their families. No members of the public were directly involved in the study design, outcome measures, analysis of data, or interpretation of study results.

## Results

Overall, 580 819 deaths occurred during the first quarter of 2020 in DSP areas that were reported by 22 May 2020. In three Wuhan DSP districts, the observed all cause mortality rate (n=6698 deaths; 1147 observed *v* 735 predicted per 100 000) was 56% (rate ratio 1.56, 95% confidence interval 1.33 to 1.87) higher than the predicted rate (table 1). This was chiefly because of a more than eightfold increase in deaths from pneumonia (n=1682; 275 *v* 33 per 100 000; 8.32, 5.19 to 17.02), including not only covid-19 related pneumonia (n=1178; 193 per 100 000) but also unspecified viral pneumonias (35 *v* 1 per 100 000) and other pneumonias. For non-covid-19 related pneumonia, most of the excess deaths occurred before mid-February 2020 (see supplementary fig 3). Apart from pneumonia, mortality rates from certain non-communicable diseases were also increased, including cardiovascular disease (n=2347; 408 *v* 316 per 100 000; 1.29, 1.05 to 1.65), particularly hypertensive heart disease (n=345; 60 *v* 30 per 100 000; 2.0, 1.24 to 4.25) and diabetes (n=262; 46 *v* 25 per 100 000; 1.83, 1.08 to 4.37). For deaths attributable to injury, the observed mortality rate was non-significantly 16% lower than the predicted rates. The mortality rates for individual types of injury differed by cause, with lower rates for road traffic incidents and higher rates for suicides and falls. When deaths from all other diseases were aggregated, the observed mortality rate was 92% higher (n=263; 46 *v* 24; 1.92, 1.17 to 4.32). The results were generally similar, albeit slightly higher, when the 2020 mortality rates were compared with the mean rates for 2015-19 that did not account for mortality trends over time (see supplementary table 4).

**Table 1:** Observed and predicted 2020 mortality rates from selected major diseases across different Disease Surveillance Point (DSP) areas

Causes of death	Wuhan				Hubei without Wuhan				China without Hubei		
	Observed per 100 000	Predicted per 100 000*	Rate ratio (95% CI)		Observed per 100 000	Predicted per 100 000*	Rate ratio (95% CI)		Observed per 100 000	Predicted per 100 000*	Rate ratio (95% CI)
All causes	1147.2	734.7	1.56 (1.33 to 1.87)		867.3	871.2	1.00 (0.86 to 1.16)		675.4	715.4	0.94 (0.86 to 1.04)
**Infectious diseases†**	290.1	47.8	6.07 (4.02 to 10.98)		27.6	31.3	0.88 (0.68 to 1.22)		20.3	29.0	0.70 (0.61 to 0.81)
Pneumonia:	275.2	33.1	8.32 (5.19 to 17.02)		11.4	15.8	0.72 (0.50 to 1.16)		9.5	17.8	0.53 (0.46 to 0.63)
Unspecified viral	34.6	0.6	56.39 (9.95 to ∞)‡		1.8	0.5	3.34 (0.96 to ∞)‡		0.4	0.9	0.43 (0.32 to 0.62)
Other	48.0	32.5	1.48 (0.92 to 3.10)		5.0	15.3	0.33 (0.23 to 0.53)		9.0	16.9	0.53 (0.46 to 0.63)
Covid-19	192.6	0.0	–		4.6	0.0	–		0.0	0.0	–
Other	14.9	16.1	0.92 (0.51 to 2.85)		16.2	15.3	1.06 (0.77 to 1.63)		10.8	11.3	0.95 (0.84 to 1.08)
**Non-communicable diseases**	757.6	625.5	1.21 (1.03 to 1.46)		744.1	760.1	0.98 (0.85 to 1.14)		606.7	635.7	0.95 (0.87 to 1.05)
Cancer	186.4	182.7	1.02 (0.81 to 1.33)		179.1	166.3	1.08 (0.94 to 1.25)		153.9	155.1	0.99 (0.93 to 1.06)
Cardiovascular disease:	408.1	316.0	1.29 (1.05 to 1.65)		416.1	424.8	0.98 (0.83 to 1.18)		334.0	349.8	0.95 (0.86 to 1.07)
Myocardial infarction	139.9	114.0	1.23 (0.93 to 1.73)		150.4	157.6	0.95 (0.78 to 1.20)		135.9	145.9	0.93 (0.84 to 1.04)
Ischaemic stroke	96.9	77.3	1.24 (0.83 to 2.19)		70.0	64.0	1.03 (0.84 to 1.30)		82.2	85.2	0.96 (0.86 to 1.07)
Haemorrhagic stroke	56.6	45.7	1.25 (0.91 to 1.91)		90.8	88.5	1.09 (0.88 to 1.42)		58.5	61.0	0.96 (0.87 to 1.08)
Hypertensive heart disease	59.9	29.9	2.00 (1.24 to 4.25)		48.3	58.7	0.82 (0.63 to 1.13)		24.4	25.6	0.95 (0.84 to 1.10)
Chronic respiratory disease:	55.6	51.8	1.07 (0.72 to 1.87)		73.4	94.9	0.77 (0.60 to 1.04)		55.2	67.2	0.82 (0.71 to 0.96)
Chronic obstructive pulmonary disease	45.5	40.5	1.13 (0.73 to 2.15)		64.9	86.8	0.75 (0.58 to 1.01)		50.2	61.6	0.82 (0.71 to 0.95)
Diabetes	45.6	24.9	1.83 (1.08 to 4.37)		17.5	17.7	0.99 (0.72 to 1.49)		18.5	19.6	0.94 (0.84 to 1.07)
Chronic kidney disease	7.2	4.8	1.51 (0.60 to ∞)‡		8.5	7.8	1.10 (0.70 to 2.16)		4.3	4.8	0.89 (0.76 to 1.07)
Other	54.6	49.3	1.11 (0.77 to 1.84)		49.5	48.6	1.02 (0.84 to 1.28)		40.8	39.1	1.04 (0.95 to 1.15)
**Injury**	53.7	46.2	1.16 (0.77 to 2.03)		83.6	74.8	1.12 (0.88 to 1.47)		37.7	40.9	0.92 (0.84 to 1.02)
Road traffic incident	4.6	7.2	0.63 (0.28 to ∞)‡		9.4	15.0	0.63 (0.44 to 0.99)		8.7	11.3	0.77 (0.68 to 0.88)
Suicide	11.7	7.0	1.66 (0.74 to ∞)†		32.2	19.9	1.62 (1.15 to 2.53)		5.3	4.9	1.09 (0.93 to 1.30)
Fall	24.5	17.1	1.43 (0.81 to 3.92)		22.0	19.0	1.16 (0.83 to 1.77)		12.4	12.8	0.97 (0.87 to 1.09)
Other	13.0	15.1	0.86 (0.45 to 2.87)		20.0	20.8	0.96 (0.64 to 1.64)		11.3	12.3	0.92 (0.77 to 1.11)
**All other diseases**	45.8	23.8	1.92 (1.17 to 4.32)		11.9	9.0	1.33 (0.86 to 2.48)		10.8	10.9	0.99 (0.86 to 1.15)

* Sum of rates from different diseases might not add up to the category sum or overall total owing to rounding in the models.

† Includes a small number of deaths from maternal, perinatal, and nutritional diseases.

‡ For certain less common diseases, the upper limits of the rate ratio become infinitely large because they involved zero predicted cases in certain circumstances.

In other parts of Hubei, the observed overall mortality rates were comparable with the predicted rates (table 1). Although there were excess deaths from covid-19 related pneumonia (n=133) as well as from unspecified viral pneumonia, the observed mortality rates from all pneumonia were non-significantly 28% lower than the predicted rates; chiefly as a result of the lower rates for non-viral pneumonia (0.33, 0.23 to 0.53). The results were similar when the 2020 rates were compared with the mean rates for 2015-19 (see supplementary table 4). As in Wuhan DSP areas, the observed mortality rates from road traffic incidents were significantly lower than predicted, whereas the rates from suicide were significantly higher than predicted.

Outside of Hubei province, the observed overall mortality rate was non-significantly 6% lower than the predicted rate (675 *v* 715 per 100 000; table 1). For a few specific diseases the observed mortality rates were significantly lower than the predicted rates, including for total pneumonia (10 *v* 18 per 100 000; 0.53, 0.46 to 0.63), chronic obstructive pulmonary disease (50 *v* 62 per 100 000; 0.82, 0.71 to 0.95), and road traffic incidents (9 *v* 11 per 100 000; 0.77, 0.68 to 0.88). For pneumonia, with the exception of a small number of deaths from covid-19 related pneumonia (n=49), significant reductions were found in deaths from unspecified viral pneumonia (0.43, 0.32 to 0.62) and non-viral pneumonia (0.53, 0.46 to 0.63), with similar findings when the 2020 data were compared with the mean rates for 2015-19 (see supplementary table 4).

**Fig 1:**
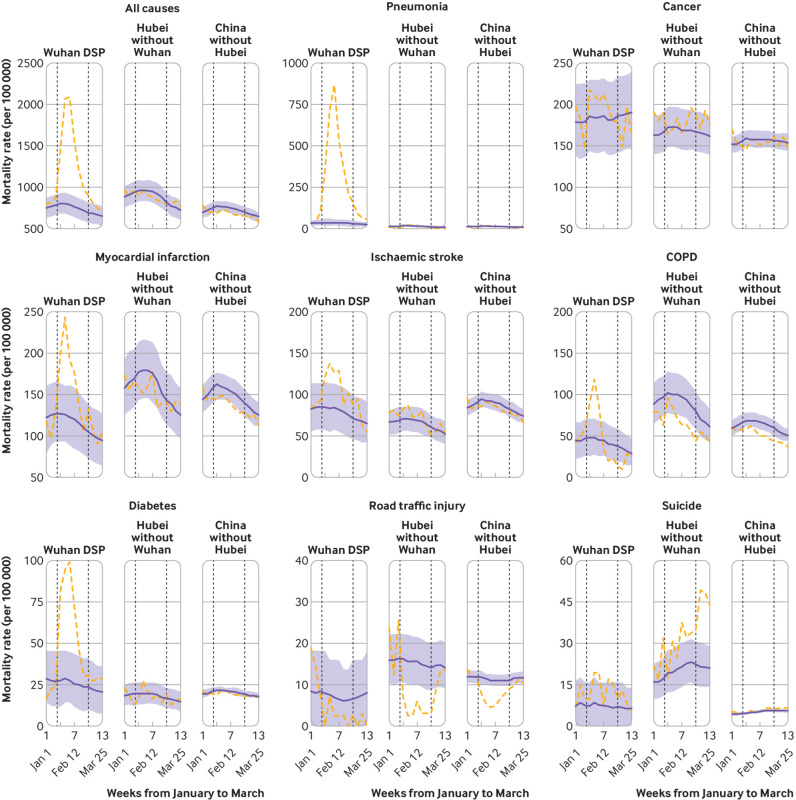
Trends in weekly observed (dashed orange lines) versus predicted (blue solid lines) mortality rates for selected major diseases between 1 January and 31 March 2020 in China across different Disease Surveillance Point areas. First vertical dotted line indicates the time when lockdown was implemented in Wuhan. Second vertical dotted line indicates when temporary makeshift hospitals were closed in Wuhan that provided central quarantines for all people who tested positive for severe acute respiratory coronavirus 2 during the coronavirus disease 2019 outbreak. For different diseases, the ranges of mortality rates vary in y axis. The shaded areas indicate 95% confidence intervals for predicted mortality rates. COPD=chronic obstructive pulmonary disease

In Wuhan DSP areas, in addition to the rapid increase followed by rapid decrease in deaths from pneumonia, similar, albeit more modest, increases were found in deaths from myocardial infarction, ischaemic stroke, chronic obstructive pulmonary disease, and diabetes (fig 1). In other parts of Hubei or other regions of China, the observed weekly mortality rates did not differ significantly from the predicted rates during January-March 2020, except for lower mortality rates from chronic obstructive pulmonary disease and road traffic incidents during the lockdown period. For pneumonia, when the figures were plotted separately from those for Wuhan using different scales, a small albeit significant increase was found in mortality during February in other parts of Hubei, mimicking, albeit to a lesser extent, that observed in Wuhan (see supplementary fig 4). In other regions of China, however, the weekly mortality rates for pneumonia were consistently lower than the mean rates in 2015-19 throughout the entire first quarter of 2020, with somewhat greater differences during than before or after the lockdown (see supplementary fig 4).

**Fig 2:**
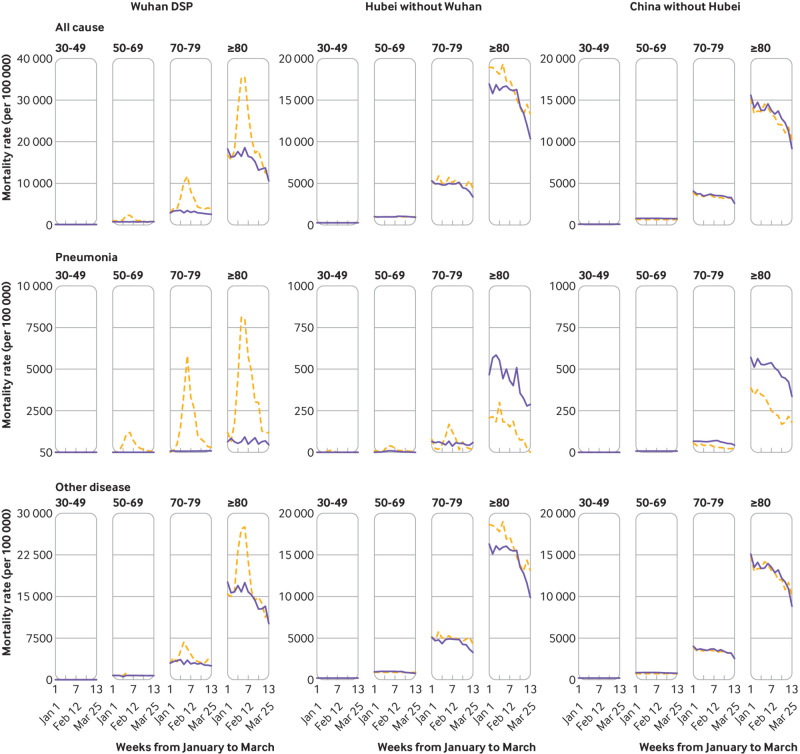
Age specific trends in weekly all cause, pneumonia, and other disease mortality rates during 1 January and 31 March 2020 compared with 2015-19 in China across different Disease Surveillance Point (DSP) areas. Dashed orange lines indicate observed mortality rates in 2020 and blue solid lines indicate mean mortality rates in 2015-19. For different diseases, the ranges of mortality rates in the y axis differ between Wuhan DSP areas and elsewhere

The rising trends in overall mortality rates in Wuhan DSP areas were chiefly observed among people aged 50 years or older, driven not only by pneumonia but also by other diseases (fig 2 and supplementary fig 5). In other Hubei areas, the weekly pneumonia mortality rates appeared consistently lower among those aged 80 years or older (fig 2), while there were smaller increases among those younger than 80 years (see supplementary fig 5). Outside of Hubei, the mortality rates for pneumonia overall appeared consistently lower among those aged 50 years or older, with little difference in mortality rates from other diseases.

In the whole of Wuhan city (ie, three DSP districts and 10 non-DSP districts), 5954 additional deaths occurred during January-March 2020 than in the same period of 2019, representing a 33% increase in overall mortality rate (table 2). (If the Wuhan DSP adjustment ratio is applied for possible delay in reporting deaths, then the increase in overall mortality in 2020 was 42%.) Among the total excess deaths, 61% (n=3653/5954) were due to covid-19 related pneumonia, 15% (920/5954) to other types of pneumonia (mainly unspecified viral pneumonia), and the remainder (1381/5954, 23%) to a range of other diseases (cardiovascular disease and hypertensive heart disease in particular, and diabetes). As in the Wuhan DSP areas, much of the increase in non-covid-19 related pneumonia occurred before mid-February 2020 (see supplementary fig 3). A significant decrease in deaths from road traffic incidents and a significant increase in deaths from suicide and falls were found (table 2).

**Table 2:** Number of observed deaths from major diseases during the first quarter of 2019 and 2020 in central and suburban districts of Wuhan city

Causes of death	Central districts (n=6)				Suburban districts (n=7)				All Wuhan city (n=13)		
	2020	2019	Ratio		2020	2019	Ratio		2020	2019	Ratio
All causes	13 767	9181	1.50		10 415	9047	1.15		24 182	18 228	1.33
Infectious diseases*	4197	781	5.37		1490	387	3.85		5687	1168	4.87
Pneumonia:	4022	539	7.46		1302	212	6.14		5324	751	7.09
Unspecified viral	500	6	83.33		191	15	12.73		691	21	32.90
Other	797	533	1.50		183	197	0.93		980	730	1.34
Covid-19	2725	0	–		928	0	–		3653	0	–
Other	175	242	0.72		188	175	1.07		363	417	0.87
**Non-communicable diseases**	8553	7623	1.12		8106	7924	1.02		16 659	15 547	1.07
Cancer	2010	2124	0.95		2223	2046	1.09		4233	4170	1.02
Cardiovascular disease:	4628	3886	1.19		4500	4379	1.03		9128	8265	1.10
Myocardial infarction	1668	1426	1.17		1313	1411	0.93		2981	2837	1.05
Ischaemic stroke	1142	938	1.22		928	867	1.07		2070	1805	1.15
Haemorrhagic stroke	512	529	0.97		978	972	1.01		1490	1501	0.99
Hypertensive heart disease	681	376	1.81		404	309	1.31		1085	685	1.58
Chronic respiratory disease:	582	618	0.94		515	715	0.72		1097	1333	0.82
Chronic obstructive pulmonary disease	479	499	0.96		361	550	0.66		840	1049	0.80
Diabetes	544	287	1.90		236	230	1.03		780	517	1.51
Chronic kidney disease	91	59	1.54		96	82	1.17		187	141	1.33
Other	698	649	1.08		536	472	1.14		1234	1121	1.10
**Injury**	527	476	1.11		571	589	0.97		1098	1065	1.03
Road traffic incident	29	97	0.30		61	153	0.40		90	250	0.36
Suicide	118	69	1.71		113	60	1.88		231	129	1.79
Fall	281	183	1.54		252	217	1.16		533	400	1.33
Other	99	127	0.78		145	159	0.91		244	286	0.85
**All other diseases**	490	301	1.63		248	147	1.69		738	448	1.65

* Includes a small number of deaths from maternal, perinatal, and nutritional diseases.

**Fig 3:**
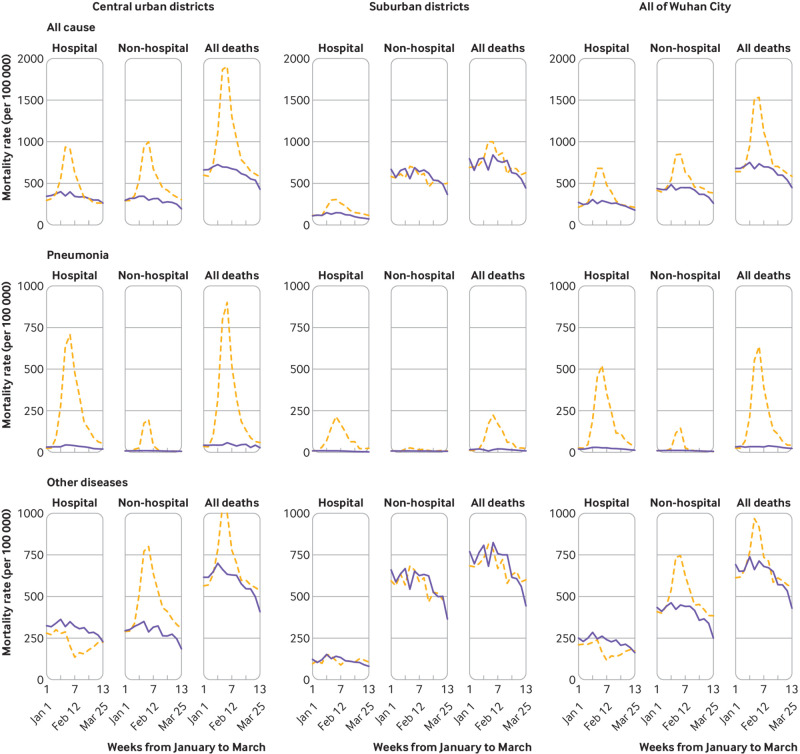
Trends in weekly hospital and non-hospital mortality rates from all causes, pneumonia, and other diseases during 1 January and 31 March 2020 compared with 2019 across different districts of Wuhan city, China. Dashed orange lines indicate observed mortality rates in 2020 and blue solid lines indicate observed mortality rates in 2019

In Wuhan city, the excess all cause mortality was substantially greater in the six central districts than in the seven suburban districts (50% *v* 15%; table 2), as were changes in weekly overall mortality rates (see supplementary fig 6). Unlike in the central districts, little difference was found in the weekly mortality from other non-pneumonia diseases between 2020 and 2019 in suburban districts. In both central and suburban districts, the excess mortality was greater in men than in women, especially for pneumonia (see supplementary fig 7). For deaths from pneumonia, the increase in 2020 was seen both inside and outside of hospital (fig 3). For deaths from causes other than pneumonia, however, there were contrasting patterns, with significant reductions in hospital deaths and significant increases in non-hospital deaths in 2020 compared with 2019. The contrasting patterns were evident only in central districts, with little difference observed in suburban districts.

## Discussion

Using nationally representative mortality data that covered one fifth of the total population in China and the whole of Wuhan city, the present study showed that during three months of the covid-19 outbreak (January to March 2020), deaths in Wuhan significantly increased not only from pneumonia, mostly related to covid-19, but also from several other major diseases. The excess mortality rates were greater in central than suburban districts and coincided with the increase and decrease of the covid-19 epidemic. Outside of Wuhan, however, no increase was found in overall mortality and, if anything, the observed mortality rates from various types of pneumonia (except that caused by SARS-CoV-2), chronic respiratory diseases, and road traffic incidents were lower than the predicated rates, all of which coincided closely with the lockdown.

### Comparison with other studies

In Wuhan city, the most recent official figures released on 17 April 2020 suggested that 3869 deaths were directly attributable to covid-19.^13^ ^20^ ^24^ Based on more careful and comprehensive analyses, our study indicated that during three months of the covid-19 outbreak, about 6000 more deaths occurred in January to March 2020 compared with the same period in 2019, including 3653 deaths from covid-19 related pneumonia and 920 excess deaths from other types of pneumonia, primarily unspecified viral pneumonia. Most of the excess deaths from non-covid-19 related pneumonia occurred before mid-February (ie, before testing for covid-19 became widely available), suggesting a high proportion of the deaths could be due to covid-19, which would increase the total estimated number of covid-19 deaths to around 4500. Apart from pneumonia, about 1400 additional deaths were due to several chronic diseases. When examined separately by location of deaths, significant reductions were found in hospital deaths and significant increases in non-hospital deaths, suggesting that difficulty in accessing hospital services or a reluctance to seek hospital care during the outbreak probably explained much of the observed excess mortality from non-pneumonia related diseases. Coinciding with the initial spread of covid-19 in Wuhan, much of the excess mortality was restricted to central districts, with much smaller numbers of excess deaths in suburban districts of Wuhan (and other parts of Hubei province).

Consistent with previous reports in China and elsewhere,^2^ ^3^ ^25^ ^26^ the excess deaths from covid-19 related and other pneumonias in Wuhan city were significantly greater in older than in younger adults, especially among those aged 70 years or older. Moreover, given age, the excess risks were greater among men than among women, consistent with previous findings in China and elsewhere.^27^ ^28^ ^29^ The reasons for differences in excess risks between men and women cannot be properly explored in the present study, which could reflect lower risks of infection, decreased susceptibility to SARS-CoV-2, or better survival prognosis after infection among women. In China, the prevalence of tobacco smoking is much higher in men than in women.^29^ Whether this might explain some of the differences in mortality between men and women warrants further investigation.

In China, much of the primary healthcare activities related to long term management of chronic diseases (eg, diabetes, hypertension, and ischaemic heart disease) is mainly undertaken through hospitals.^15^ During the outbreak, access to essential lifesaving drugs such as insulin, and acute procedures such as stenting, or the ability to monitor and manage major diseases and associated risk factors (eg, blood pressure, blood glucose levels) through hospital visits was severely compromised, leading to an exacerbation of established diseases and an increased risk of deaths (eg, diabetic crisis). Evidence also suggests that covid-19 might cause microclots in the circulatory system,^30^ ^31^ and embed in the heart, resulting in myocarditis,^30^ ^32^ both of which, if true, could increase the risk of cardiovascular diseases such as myocardial infarction and ischaemic stroke. Similarly, it has been hypothesised that SARS-CoV-2 might affect the pancreas and thereby increase the risk of new onset diabetes, as well as cause exacerbation of existing diabetes, leading to increased risk of deaths.^33^ The excess mortality rates were particularly high for hypertensive heart disease, which could be due to poor control of hypertension during the lockdown and possible interaction of SARS-CoV-2 infection with certain antihypertensive drugs, such as angiotensin converting enzyme inhibitors. Alternatively, this might be partly or wholly due to coding issues. Indeed, in Wuhan city, 13% of all deaths attributed to hypertensive heart disease during 2020 (i.e., coded as underlying cause of deaths on death certificates) were also found to have myocardial infarction, coronary heart disease, or sudden cardiac death as contributing causes. Although no evidence of immediate excess mortality from cancer was found, the long term adverse impacts of the covid-19 outbreak on cancer survival (and other major diseases) cannot be excluded owing to delayed diagnosis.

Outside of Wuhan city in Hubei and other regions of China, the situation is totally different. Although a small increase in deaths from covid-19 related pneumonia occurred, these were more than offset by a reduction in deaths from other types of pneumonia, which was particularly evident after the lockdown. Likewise, there was also a reduction in mortality from chronic respiratory diseases, mainly chronic obstructive pulmonary disease, consistent with reduced respiratory infections that could otherwise exacerbate chronic obstructive pulmonary disease to cause death. It is possible that the lockdown along with improved personal hygiene, such as wearing face masks and regular hand washing, might reduce the risks of transmission of other pathogens that cause pneumonia. Consistent with our study findings, the 2019-20 winter influenza season in Hong Kong was much shorter than that during 2015-19, resulting in a more than 60% decrease in the number of deaths from laboratory confirmed influenza in adults.^34^ During lockdown all unnecessary travel was suspended, leading to an expected reduction in deaths from road traffic incidents; however, as in Wuhan city, significant excess deaths occurred from suicide in Hubei (but not in other regions of China), perhaps owing to poor mental wellbeing associated with the covid-19 outbreak, a stricter lockdown in Hubei province than elsewhere in China, and inadequate social support for mental wellbeing. Although the proportional excess risks were similar (around 60%), the absolute increases in suicide mortality were much greater in other parts of Hubei than in Wuhan, reflecting a higher burden of mental disorders in resource poor rural areas outside of Wuhan city. Consistent with our study findings, a recent study in China also showed that mental health symptoms were common during the covid-19 outbreak, especially among residents in Hubei and those with confirmed or suspected infection or those who had contact with someone with covid-19.^35^

### Strengths and limitations of this study

The chief strengths of the present study include the large sample size, the use of nationally representative samples covering all regions of mainland China, well characterised mortality data (eg, <5% with ill defined causes of death), the ability to examine and compare mortality data between 2020 and 2015-19, and the availability of cause specific mortality data in Wuhan city beyond the three DSP districts. However, our study also has several limitations. Firstly, individual data collected through routine death registries were too limited to enable more detailed analyses by certain risk factors (eg, smoking status, adiposity) and disease histories (eg, hypertension, diabetes, coronary heart disease). Secondly, although appropriate, the applied adjustments for probable delay in reporting death across different DSP areas could still result in under-estimation or overestimation of the likely excess mortality, especially in Wuhan city and for non-pneumonia related deaths. However, the consistent results for excess mortality from Wuhan DSP calculated based on trend over a prolonged period and from Wuhan central districts based on 2020 and 2019 comparisons indicated that any underreporting for the whole of Wuhan city is likely to be small. Thirdly, without proper linkages to covid-19 test results, it is difficult to reliably assess the true excess mortality attributed directly or indirectly to covid-19. Moreover, there are challenges in determining and assigning a single underlying cause of death, especially during the early stages of the outbreak and among patients with multiple pre-existing diseases, as is the case with hypertensive heart disease. However, any misclassifications might only affect risk estimates for specific diseases and not overall mortality. Fourthly, although nationally representative, the DSP system currently covers only one fifth of the total population, and for specific causes of death our ability to reliably detect modest changes over time was still limited because of relatively small number of events in different areas. Fifthly, we were not able to directly examine the impact of the covid-19 outbreak on hospital admissions, routine clinical examinations (eg, for cancer diagnosis), and case fatality rates, as the DSP system does not routinely collect such information.

### Conclusion

Nationwide evidence about trends in total and cause specific mortality during three months of the initial outbreak of covid-19 in China shows that in Wuhan city (especially in central districts where the outbreak was particularly serious), the death rate from all causes was about 50% higher during the first quarter of 2020, with the excess mortality due to not only pneumonia but also several other major diseases. Outside of Wuhan, no evidence was found of any significant increase in overall mortality, suggesting the success of the rapid control of the spread of SARS-CoV-2 in addition to appropriate maintenance of healthcare services during the nationwide lockdown. Moreover, the lockdown and the associated behavioural changes (eg, wearing face masks, regular hand washing, social distancing, and restricted travel) also seemed to have other unintended health benefits in addition to the intended effects of reducing the spread of SARS-CoV-2. These findings call for the establishment of unified nationwide death registries that cover the entire population of China. Furthermore, the findings highlight the need for rapid and coordinated actions during major outbreaks of infectious diseases to contain, suppress, and eradicate transmission and minimise detrimental effects on human health and societal and economic activities.

**What is already known on this topic**

In China the major outbreak of covid-19 that started in Wuhan city, Hubei province during late December 2019 led to a nationwide lockdown during late January 2020, which was subsequently lifted in early April 2020
Although various estimates have been made about the number of covid-19 related deaths in Wuhan city and elsewhere, no study has systematically examined the overall and cause specific mortality across different parts of China during the three months of the covid-19 outbreak

**What this study adds**

In this nationally representative study covering more than 300 million people, Wuhan city experienced significant excess deaths not only from pneumonia, chiefly covid-19 related, but also from several other major diseases
Outside of Wuhan city, overall mortality did not increase, and, if anything, the observed mortality rates from various types of pneumonia (except that caused by severe acute respiratory syndrome coronavirus 2), chronic respiratory diseases, and road traffic incidents were lower than the predicated rates, all of which coincided closely with the lockdown

## Web extra

Extra material supplied by authors

## Notes

Contributors: JL, LZ, and YY contributed equally to the paper as joint first authors. MZ (zhoumaigeng@ncncd.chinacdc.cn) and ZC (zhengming.chen@ndph.ox.ac.uk) are joint corresponding authors. ZC, MZ, LW, PY, JW, LZ, and YY conceived and designed the study. JL, YZ, and WW performed the statistical analysis. JL, YZ, MZ, and ZC drafted the manuscript. All authors contributed to acquisition, analysis, or interpretation of data and revised the report and approved the final version before submission. MZ and ZC are the guarantors and attest that all listed authors meet authorship criteria and that no others meeting the criteria have been omitted.

Ethical approval: Not required.

Data sharing: The mortality data can be applied for through a government data sharing portal (www.phsciencedata.cn/Share/edtShare.jsp).

The lead author (MZ) affirms that the manuscript is an honest, accurate, and transparent account of the study being reported; that no important aspects of the study have been omitted; and that any discrepancies from the study as planned (and, if relevant, registered) have been explained.

Dissemination to participants and related patient and public communities: The results of this study will be reported through the media centre of the authors’ institutions under authors’ supervision.

Provenance and peer review: Not commissioned; externally peer reviewed.

## References

[1] Zhu N Zhang D Wang W China Novel Coronavirus Investigating and Research Team A Novel Coronavirus from Patients with Pneumonia in China, 2019. N Engl J Med 2020;382:727-33. 10.1056/NEJMoa2001017 31978945 PMC7092803

[2] Li Q Guan X Wu P Early Transmission Dynamics in Wuhan, China, of Novel Coronavirus-Infected Pneumonia. N Engl J Med 2020;382:1199-207. 10.1056/NEJMoa2001316 31995857 PMC7121484

[3] Verity R Okell LC Dorigatti I Estimates of the severity of coronavirus disease 2019: a model-based analysis. Lancet Infect Dis 2020;20:669-77. 10.1016/S1473-3099(20)30243-7 32240634 PMC7158570

[4] The Novel Coronavirus Pneumonia Emergency Response Epidemiology Team [The epidemiological characteristics of an outbreak of 2019 novel coronavirus iseases (COVID-19) in China.] China CDC Weekly 2020;2(8):113-22 10.46234/ccdcw2020.032. PMC8392929 34594836

[5] Munster VJ Koopmans M van Doremalen N van Riel D de Wit E A Novel Coronavirus Emerging in China - Key Questions for Impact Assessment. N Engl J Med 2020;382:692-4. 10.1056/NEJMp2000929 31978293

[6] Pan A Liu L Wang C Association of Public Health Interventions With the Epidemiology of the COVID-19 Outbreak in Wuhan, China. JAMA 2020;323:1915-23. 10.1001/jama.2020.6130 32275295 PMC7149375

[7] Zhang J Litvinova M Wang W Evolving epidemiology and transmission dynamics of coronavirus disease 2019 outside Hubei province, China: a descriptive and modelling study. Lancet Infect Dis 2020;20:793-802. 10.1016/S1473-3099(20)30230-9 32247326 PMC7269887

[8] World Health Organization. Coronavirus Disease (COVID-19) Situation Report–161. 29 June 2020. https://www.who.int/docs/default-source/coronaviruse/situation-reports/20200629-covid-19-sitrep-161.pdf?sfvrsn=74fde64e_2

[9] Liu S Wu X Lopez AD An integrated national mortality surveillance system for death registration and mortality surveillance, China. Bull World Health Organ 2016;94:46-57. 10.2471/BLT.15.153148 26769996 PMC4709796

[10] Chinazzi M Davis JT Ajelli M The effect of travel restrictions on the spread of the 2019 novel coronavirus (COVID-19) outbreak. Science 2020;368:395-400. 10.1126/science.aba9757 32144116 PMC7164386

[11] Hanlon P Chadwick F Shah A COVID-19 – exploring the implications of long-term condition type and extent of multimorbidity on years of life lost: a modelling study. Wellcome Open Res 2020;5:75 10.12688/wellcomeopenres.15849.1. PMC7927210 33709037

[12] Huang C Wang Y Li X Clinical features of patients infected with 2019 novel coronavirus in Wuhan, China. Lancet 2020;395:497-506. 10.1016/S0140-6736(20)30183-5 31986264 PMC7159299

[13] Zhou F Yu T Du R Clinical course and risk factors for mortality of adult inpatients with COVID-19 in Wuhan, China: a retrospective cohort study. Lancet 2020;395:1054-62. 10.1016/S0140-6736(20)30566-3 32171076 PMC7270627

[14] Li Z Chen Q Feng L China CDC COVID-19 Emergency Response Strategy Team Active case finding with case management: the key to tackling the COVID-19 pandemic. Lancet 2020;396:63-70. 10.1016/S0140-6736(20)31278-2 32505220 PMC7272157

[15] Chen S Zhang Z Yang J Fangcang shelter hospitals: a novel concept for responding to public health emergencies. Lancet 2020;395:1305-14. 10.1016/S0140-6736(20)30744-3 32247320 PMC7270591

[16] Yang G Hu J Rao KQ Ma J Rao C Lopez AD Mortality registration and surveillance in China: History, current situation and challenges. Popul Health Metr 2005;3:3. 10.1186/1478-7954-3-3 15769298 PMC555951

[17] Yang G [Selection of DSP points in second stage and their presentation]. Zhonghua Liu Xing Bing Xue Za Zhi 1992;13:197-201. 1301261

[18] Li L Liu Y Wu P Influenza-associated excess respiratory mortality in China, 2010-15: a population-based study. Lancet Public Health 2019;4:e473-81. 31493844 10.1016/S2468-2667(19)30163-X PMC8736690

[19] Jan H Faisal S Khan A COVID-19: Review of Epidemiology and Potential Treatments Against 2019 Novel Coronavirus. Discoveries (Craiova) 2020;8:e108. 10.15190/d.2020.5 32377559 PMC7199242

[20] Yan Y Shin WI Pang YX The First 75 Days of Novel Coronavirus (SARS-CoV-2) Outbreak: Recent Advances, Prevention, and Treatment. Int J Environ Res Public Health 2020;17:2323. 10.3390/ijerph17072323 32235575 PMC7177691

[21] Kaur I Sharma A Jakhar D Coronavirus disease (COVID-19): An updated review based on current knowledge and existing literature for dermatologists. Dermatol Ther 2020;33:e13677. 10.1111/dth.13677 32447820 PMC7283871

[22] Noufaily A Enki DG Farrington P Garthwaite P Andrews N Charlett A An improved algorithm for outbreak detection in multiple surveillance systems. Stat Med 2013;32:1206-22. 10.1002/sim.5595 22941770

[23] Meyer S, Held L, Höhle M. Spatio-Temporal Analysis of Epidemic Phenomena Using the R Package surveillance. *J Stat Softw* 2017;77. https://www.jstatsoft.org/article/view/v077i11.

[24] Wu JT Leung K Leung GM Nowcasting and forecasting the potential domestic and international spread of the 2019-nCoV outbreak originating in Wuhan, China: a modelling study. Lancet 2020;395:689-97. 10.1016/S0140-6736(20)30260-9 32014114 PMC7159271

[25] Riou J Althaus CL Pattern of early human-to-human transmission of Wuhan 2019 novel coronavirus (2019-nCoV), December 2019 to January 2020. Euro Surveill 2020;25:25. 10.2807/1560-7917.ES.2020.25.4.2000058 32019669 PMC7001239

[26] Chan JF Yuan S Kok KH A familial cluster of pneumonia associated with the 2019 novel coronavirus indicating person-to-person transmission: a study of a family cluster. Lancet 2020;395:514-23. 10.1016/S0140-6736(20)30154-9 31986261 PMC7159286

[27] Khamis RY Ammari T Mikhail GW Gender differences in coronary heart disease. Heart 2016;102:1142-9. 10.1136/heartjnl-2014-306463 27126397

[28] Guan WJ Ni ZY Hu Y China Medical Treatment Expert Group for Covid-19 Clinical Characteristics of Coronavirus Disease 2019 in China. N Engl J Med 2020;382:1708-20. 10.1056/NEJMoa2002032 32109013 PMC7092819

[29] Chen Z Peto R Zhou M China Kadoorie Biobank (CKB) collaborative group Contrasting male and female trends in tobacco-attributed mortality in China: evidence from successive nationwide prospective cohort studies. Lancet 2015;386:1447-56. 10.1016/S0140-6736(15)00340-2 26466050 PMC4691901

[30] Clerkin KJ Fried JA Raikhelkar J COVID-19 and Cardiovascular Disease. Circulation 2020;141:1648-55. 10.1161/CIRCULATIONAHA.120.046941 32200663

[31] Zhang Y Xiao M Zhang S Coagulopathy and Antiphospholipid Antibodies in Patients with Covid-19. N Engl J Med 2020;382:e38. 10.1056/NEJMc2007575 32268022 PMC7161262

[32] Akhmerov A Marbán E COVID-19 and the Heart. Circ Res 2020;126:1443-55. 10.1161/CIRCRESAHA.120.317055 32252591

[33] Bornstein SR Rubino F Khunti K Practical recommendations for the management of diabetes in patients with COVID-19. Lancet Diabetes Endocrinol 2020;8:546-50. 10.1016/S2213-8587(20)30152-2 32334646 PMC7180013

[34] Chan KH Lee PW Chan CY Lam KBH Ho PL Monitoring respiratory infections in covid-19 epidemics. BMJ 2020;369:m1628. 10.1136/bmj.m1628 32366507

[35] Shi L Lu ZA Que JY Prevalence of and Risk Factors Associated With Mental Health Symptoms Among the General Population in China During the Coronavirus Disease 2019 Pandemic. JAMA Netw Open 2020;3:e2014053. 10.1001/jamanetworkopen.2020.14053 32609353 PMC7330717

