# Supplementary material for: Excess mortality in Wuhan city and other parts of China during the three months of the covid-19 outbreak: findings from nationwide mortality registries
Source: BMJ. 2021 Feb 24;372:n415. doi: 10.1136/bmj.n415 (PMC7900645; doi:10.1136/bmj.n415)
Supplement: Supplementary file 1 — Supplementary information: additional figures 1-8 and tables 1-4 [file liuj061031.ww.pdf]

Supplemental fig 1. Distribution of DSP areas in a) China and b) Wuhan city

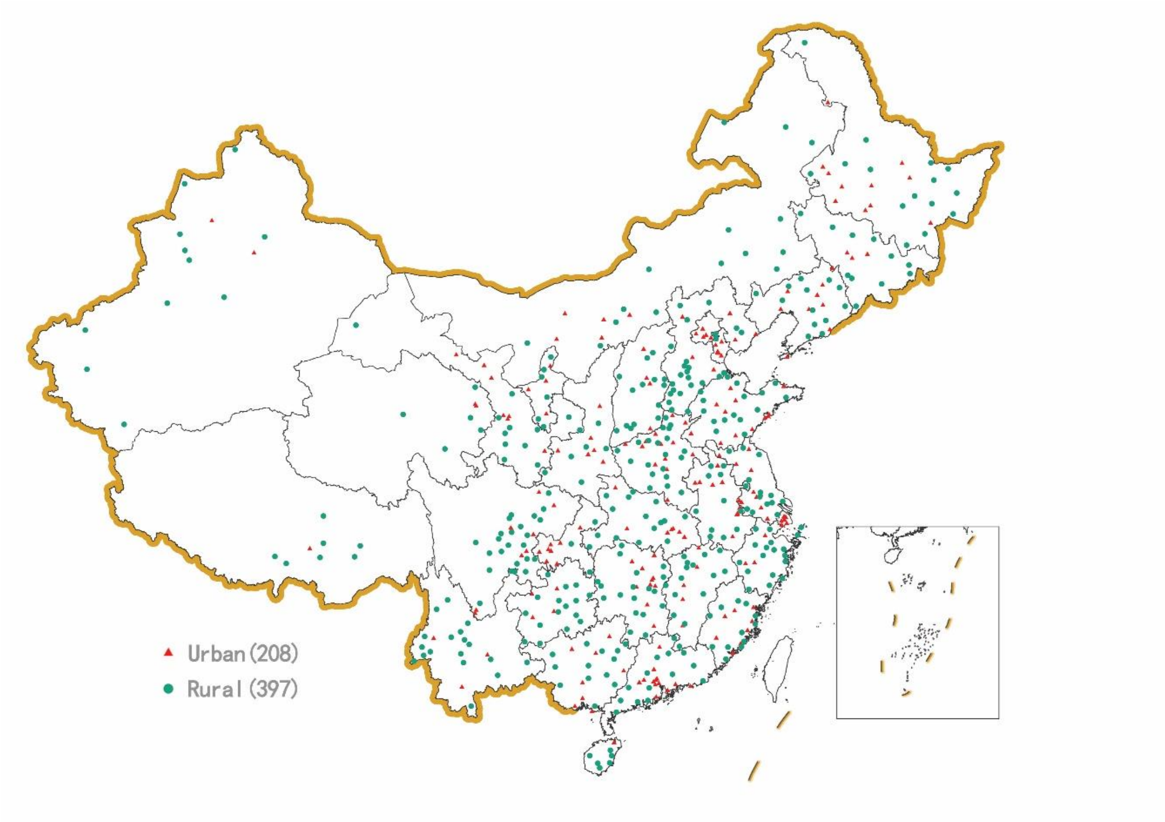

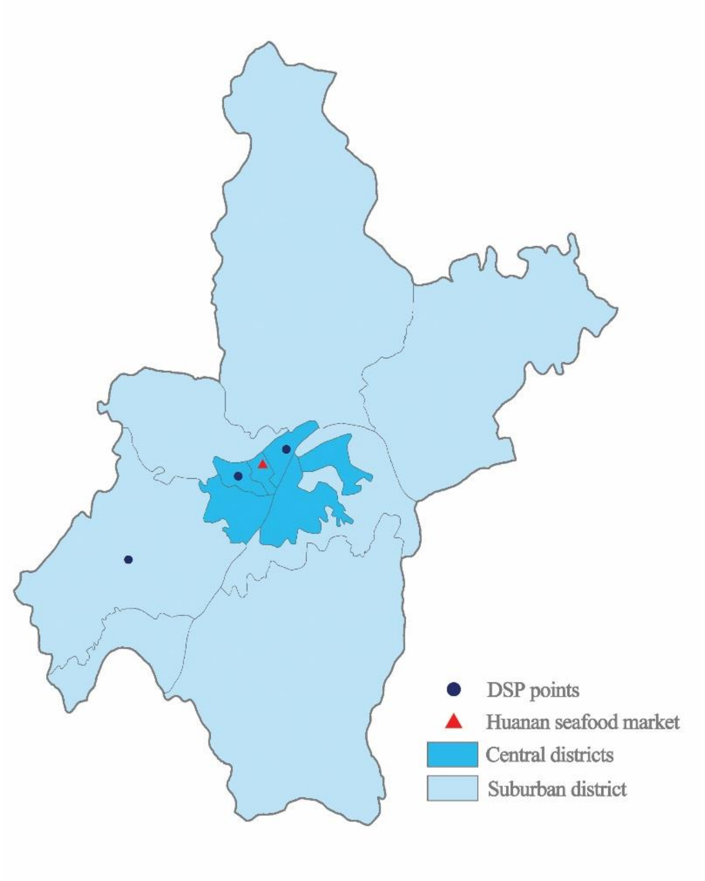

a) DSP areas across China (n=605)

b) DSP areas in Wuhan city (n=3)

Supplemental fig 2. Trends in weekly mortality rates from selected major diseases during January 2015 and March 2020 in DSP areas

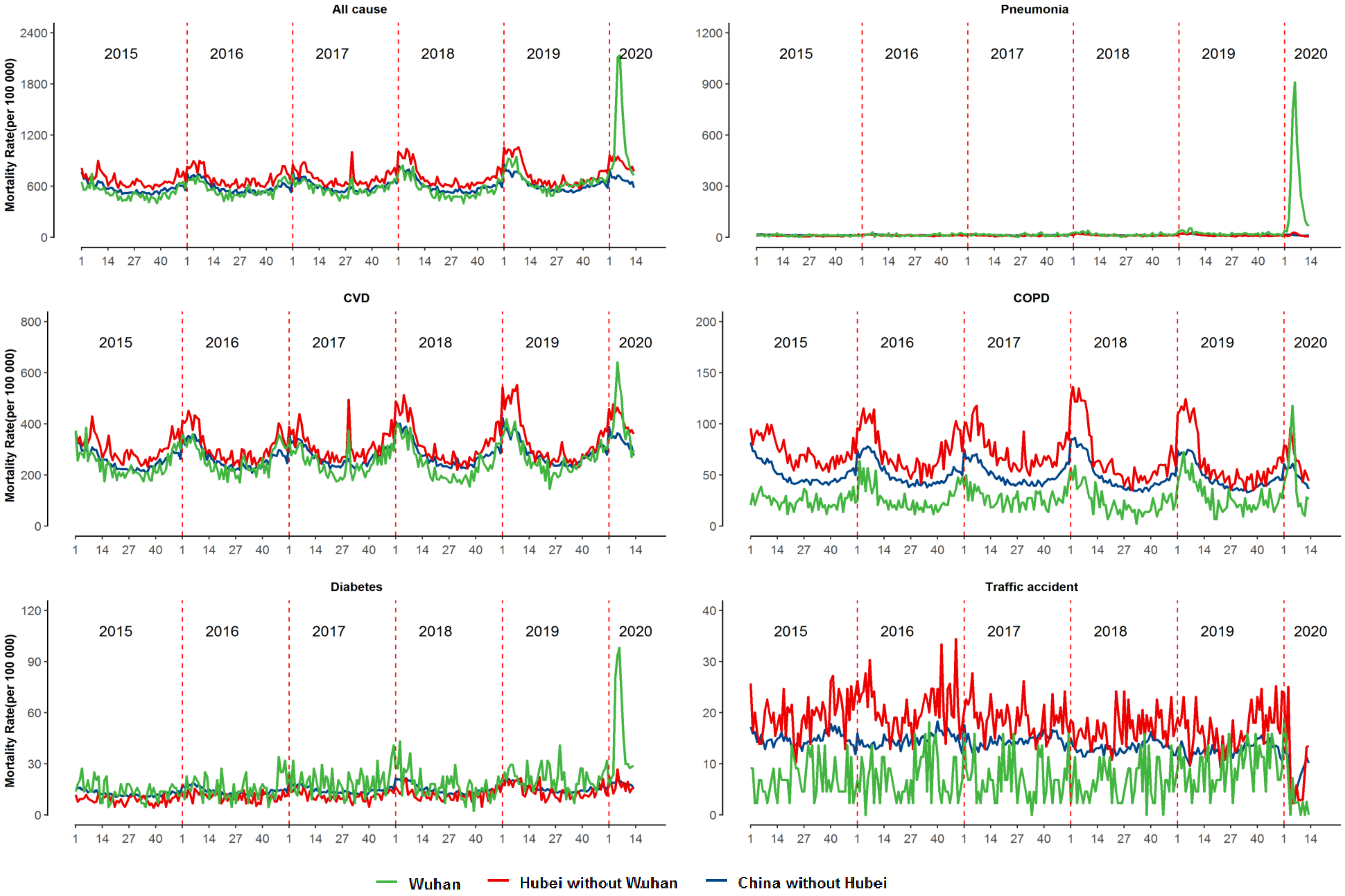

Supplemental fig 3. Distribution of deaths from different types of pneumonia by time in Wuhan, 2020 vs 2015-19

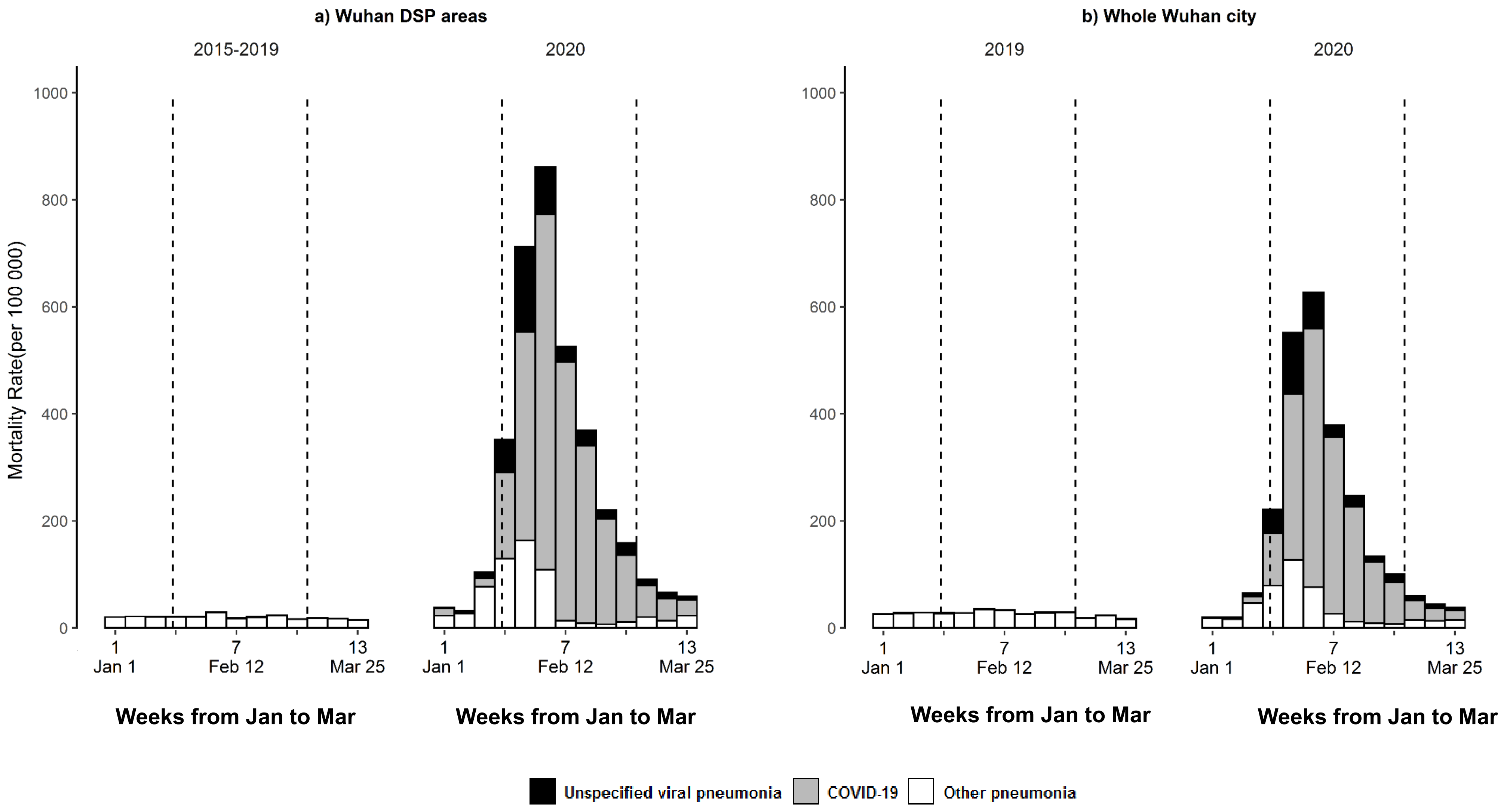

Supplemental fig 4. Trends in weekly observed (red line) versus predicted (blue line) mortality rates from pneumonia during 1 January and 31 March 2020 in different DSP areas (with different mortality scales used in Wuhan and elsewhere)

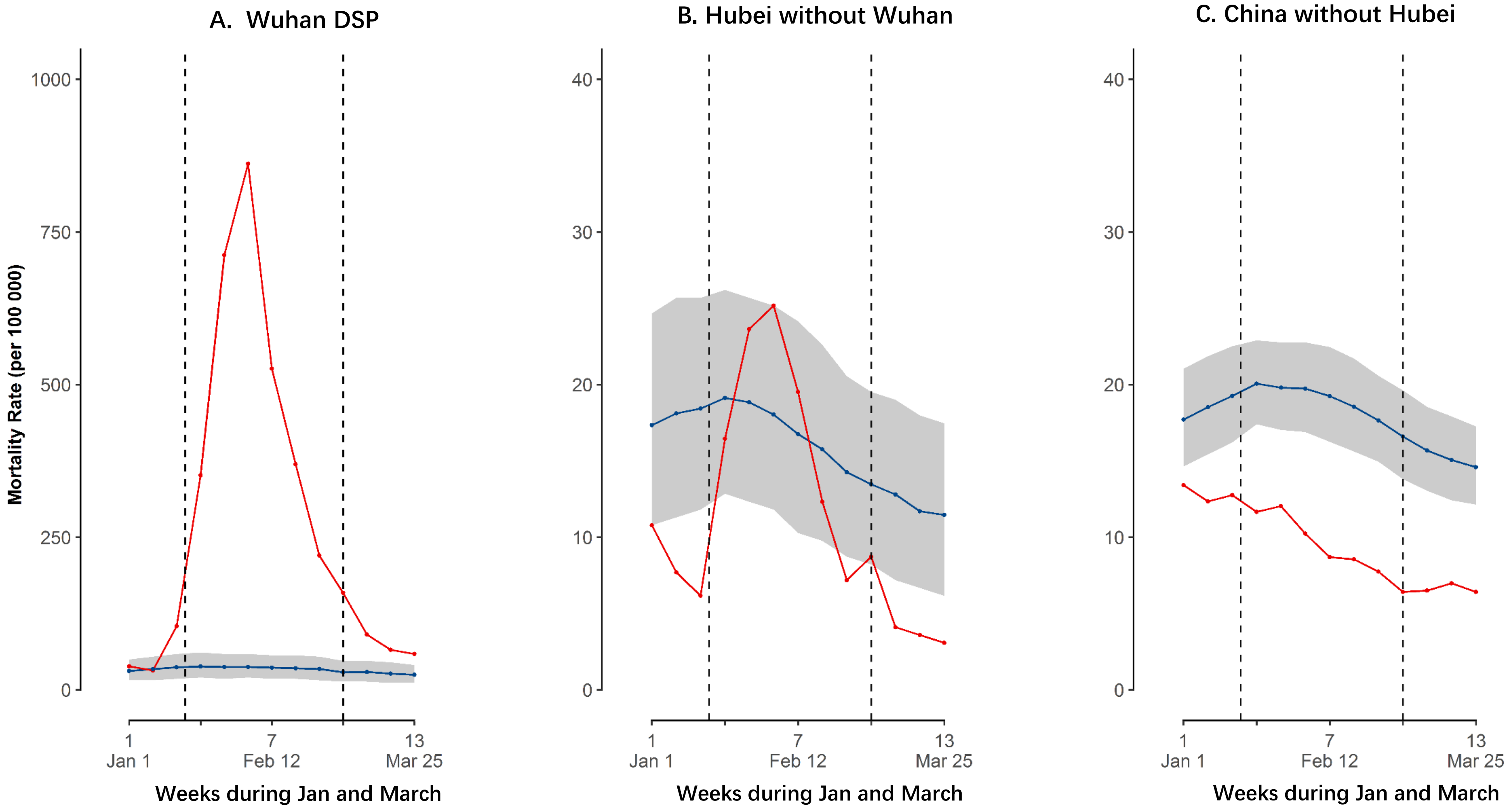

Supplemental fig 5. Trends in age-specific weekly all-cause, pneumonia and other disease mortality rates during 1 January and 31 March in 2020 (red line) versus 2015-2019 (blue line) at ages 30-69 in different DSP areas (with different mortality scales used in Wuhan and elsewhere)

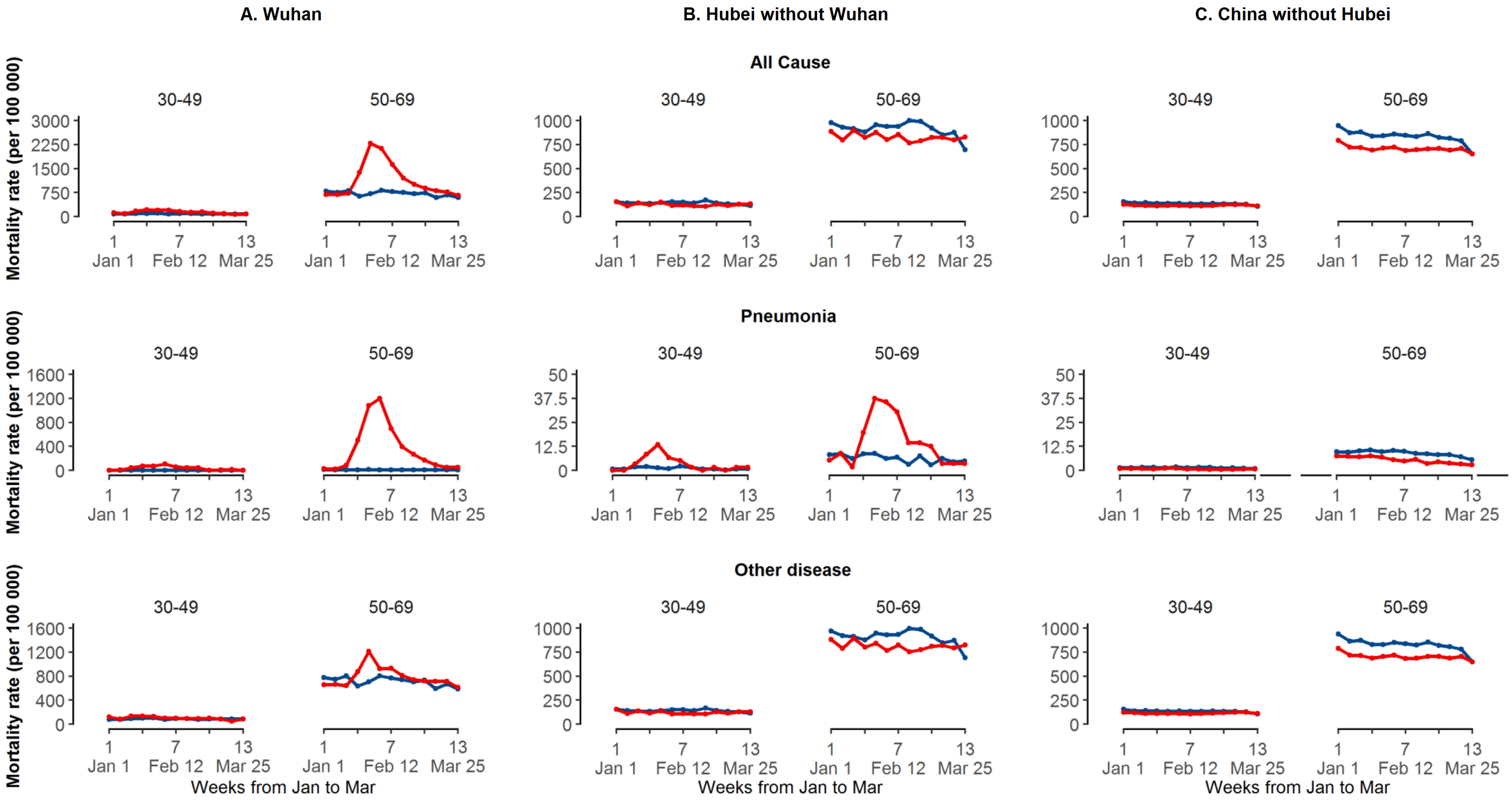

Supplemental fig 6. Trends in weekly mortality rates from pneumonia and other diseases during 1 January and 31 March in 2020 (red line) versus 2019 (blue line) in central urban and suburban districts of Wuhan city

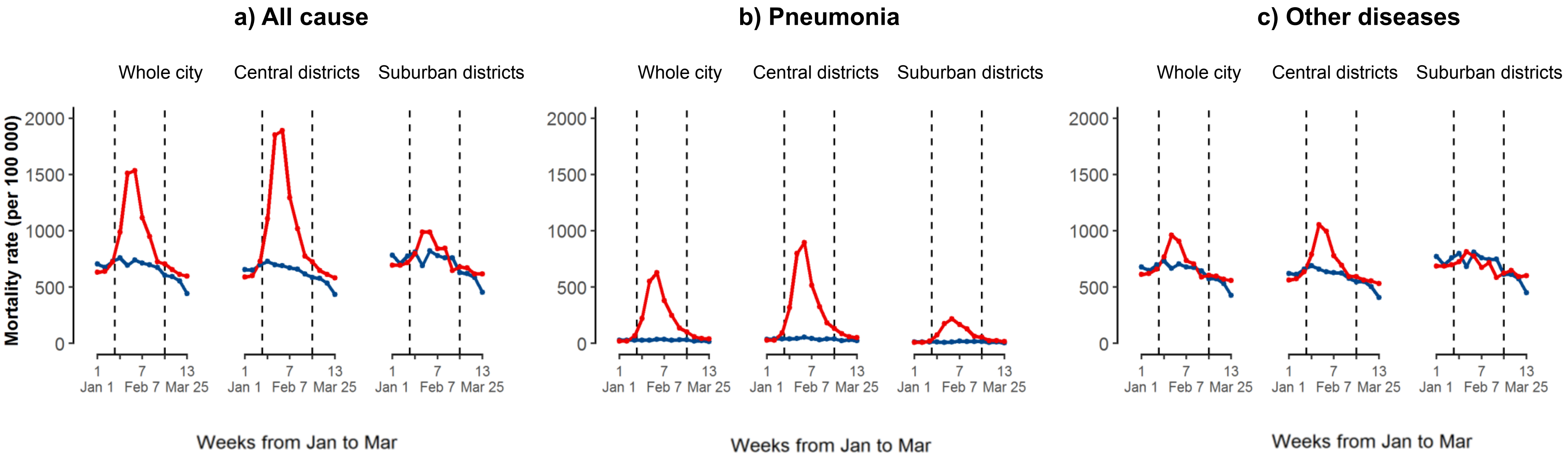

Supplemental fig 7. Trends in weekly mortality rates from pneumonia and non-pneumonia diseases during 1 January and 31 March in 2020 (red line) versus 2019 (blue line) by sex in Wuhan city

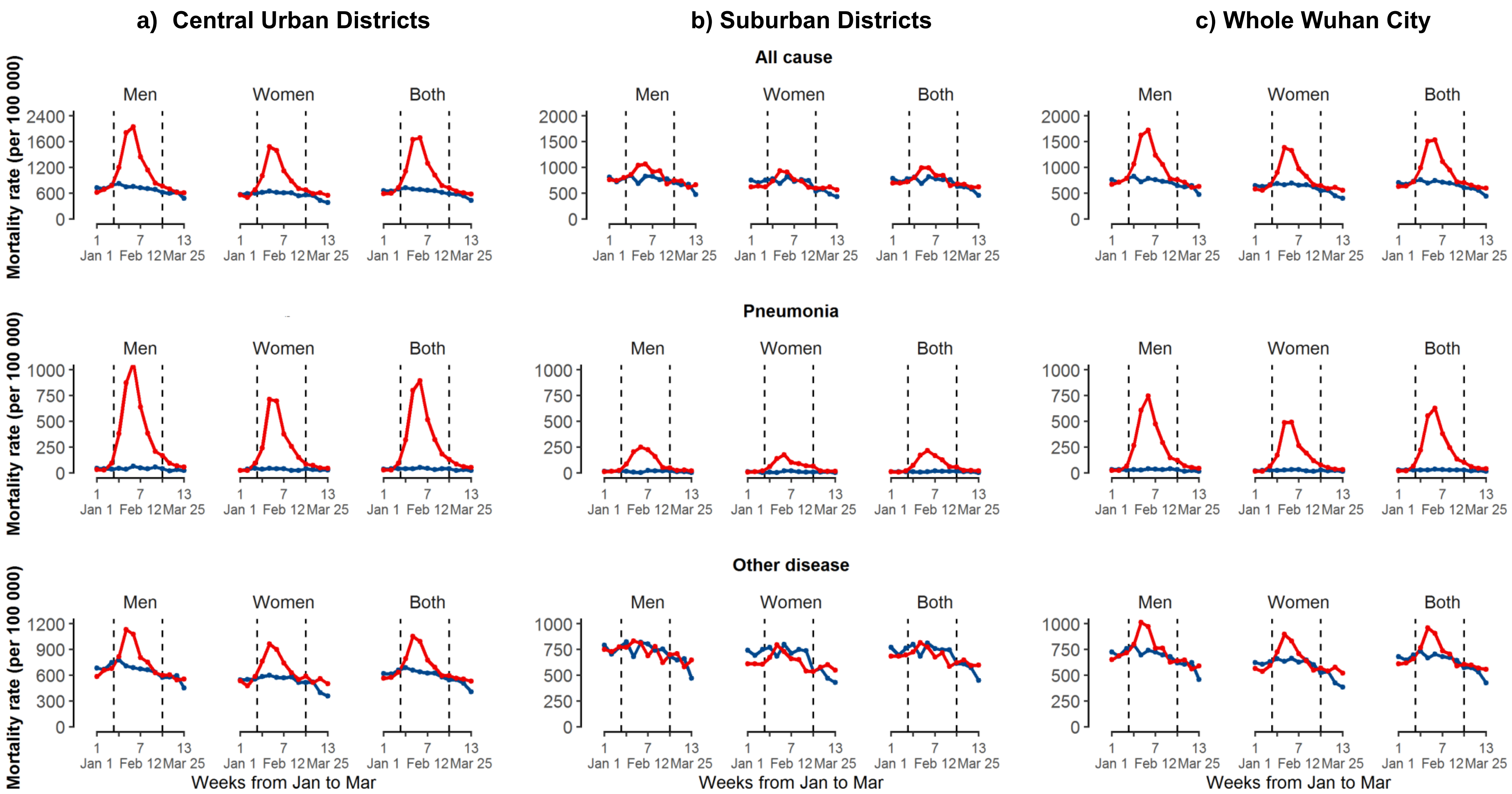

Supplemental fig 8: Trends in weekly mortality rate from selected major diseases during 1 January and 31 March in 2020 (red line) versus 2015-2019 (blue line) in different DSP areas (A. Wuhan DSP; B. Hubei without Wuhan; C. China without Hubei)

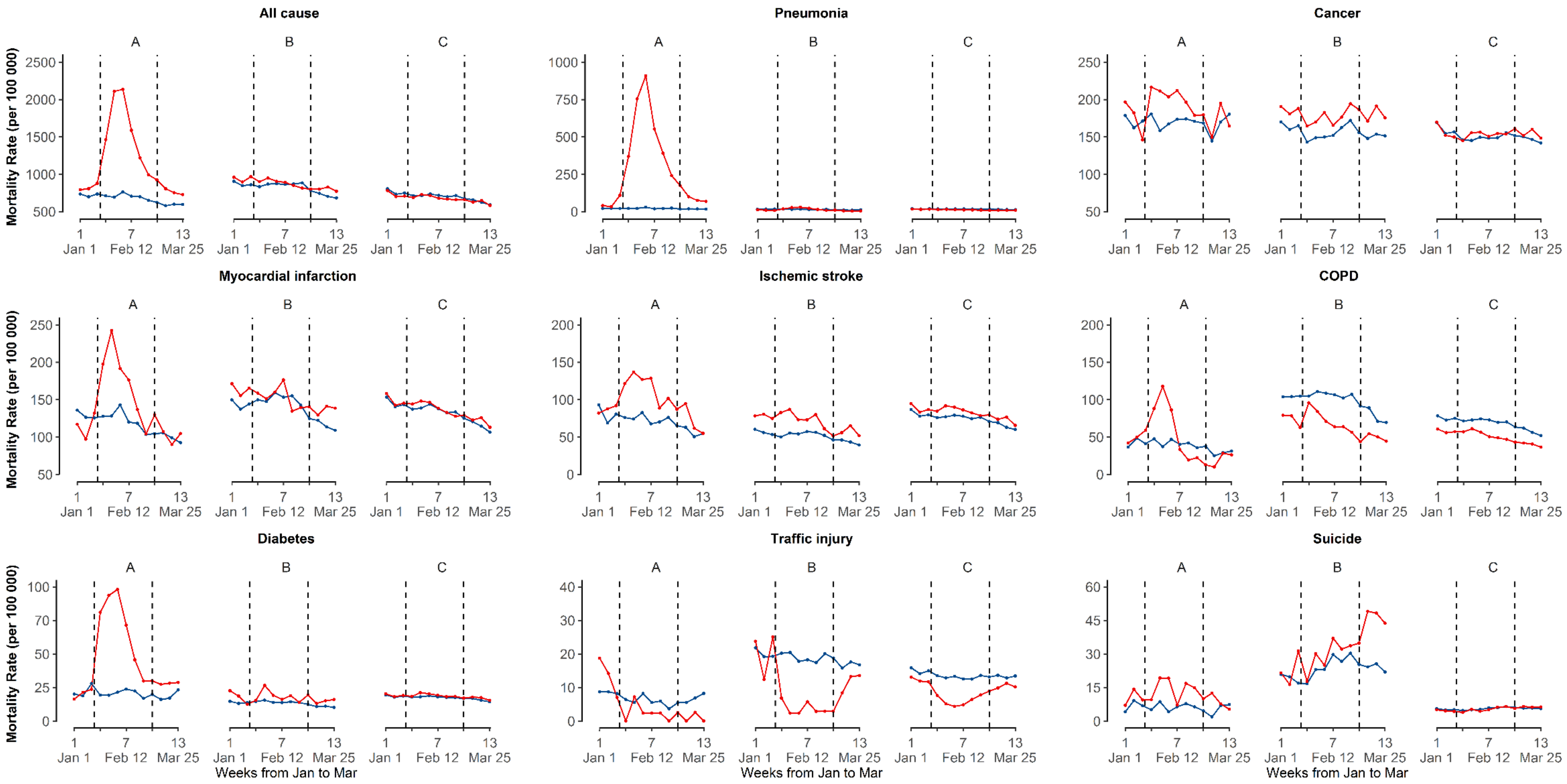

Supplemental table 1. Causes of death and associated ICD-10 codes

Causes of death	ICD-10 codes
1) **Infectious diseases** (communicable diseases plus maternal, perinatal, and nutritional conditions)	A00–B99, G00–G04, N70–N73, J00–J06, J10–J18, J20–J22, J98.4,H65–H66, O00–O99, P00–P96, E00–E02, E40–E46, E50, D50–D53, D64.9, E51–E64, U07.1
Pneumonia	J12-J18, J98.4, U07.1
Virus pneumonia (unspecified)	J12, J12.8, J12.9
Other pneumonia	J12.0-J12.7, J13-J18, J98.4
COVID-19	U07.1
Other diseases	A00–B99, G00–G04, N70–N73, J00–J06, J10–J11, J20–J22, H65–H66, O00–O99, P00–P96, E00–E02, E40–E46, E50, D50–D53, D64.9, E51–E64
**2) Chronic non-communicable diseases (NCDs)**	C00–C97, D00–D48, D55–D64 (minus D64.9), D65–D89, E03–E07, E10–E16, E20–E34, E65–E88, F01–F99, G06–G98, H00–H61, H68–H93, I00–I99, J30–J98 (minus J98.4), K00–K92, N00–N64, N75–N98, L00–L98, M00–M99, Q00–Q99
Cancer	C00–C97
Cardiovascular diseases	I00–I99
Myocardial infarction	I20–I25
Ischemic stroke	I63, I65, I66, I76.2, I67.3, I67.5, I67.6, I69.3
Hemorrhagic stroke	I60–I62, I67.0, I76.1, I96.0, I69.1, I69.2
Hypertensive heart disease	I10–I13
Chronic respiratory disease	J30–J98 (minus J98.4)
COPD	J40–J44
Chronic kidney diseases	N02-N08.8, N15.0, N18-N18.9
Diabetes	E10–E14
Other NCDs	D00–D48, D55–D64 (minus D64.9), D65–D89, E03–E07, E15-E16, E20–E34, E65–E88, F01–F99, G06–G98, H00–H61, H68–H93, K00–K92,N00-N01, N08.9,N15, N15.1 - N17.9, N19-N64, L00–L98, M00–M99, Q00–Q99
**3) Injury**	V01–Y89
Traffic accidents	V01–V04, V06, V09–V80, V87, V89, V99
Suicide	X60–X84, Y87.0
Fall	W00–W19
Other injury	V05, V08,V81-V86,V88,W20-X59, X85-Y86,Y87,Y87.1-Y89

Supplemental table 2. Number of deaths during the first quarter reported by 22 May in each year and adjustment ratios for delay in reporting across different DSP areas

		Wuhan DSP (n=3)			Hubei without Wuhan (n=19)			China without Hubei (n=583)		
Week	Starting date of the week	Mean No. of reported Deaths, 2015-19	Delay adjustment ratio (%)*	No. of reported deaths in 2020	Mean No. of reported Deaths, 2015-19	Delay adjustment ratio (%)*	No. of reported deaths in 2020	Mean No. of reported Deaths, 2015-19	Delay adjustment ratio (%)*	No. of reported deaths in 2020
1	1 Jan	321	96.94	339	1771	90.84	1697	49771	83.88	41138
2	8 Jan	305	95.87	341	1660	91.26	1589	45315	84.51	37229
3	15 Jan	321	96.28	372	1681	90.15	1699	46472	83.80	37349
4	22 Jan	310	95.27	614	1635	90.06	1577	43955	84.19	36447
5	29 Jan	302	94.46	879	1702	88.66	1638	44117	83.21	38134
6	5 Feb	332	94.73	892	1709	88.62	1562	45516	82.23	36889
7	12 Feb	308	95.20	666	1694	88.80	1541	44453	82.23	35078
8	19 Feb	305	94.54	509	1704	89.39	1478	43147	82.63	34667
9	26 Feb	284	91.43	400	1732	88.53	1405	44183	80.14	33214
10	4 Mar	272	90.95	370	1526	87.02	1354	41756	77.54	32198
11	11 Mar	252	91.11	324	1459	85.87	1336	40717	76.93	30380
12	18 Mar	261	88.37	293	1376	85.13	1375	38707	75.85	31002
13	25 Mar	228	86.81	278	1148	83.44	1252	31495	74.96	27399
**Total**	**Jan-March**	**3801**	**93.52**	**6277**	**20797**	**88.50**	**19503**	**559604**	**81.20**	**451124**

* Estimated as the mean weekly number of deaths during January-March in 2015-19 that were reported by 22 May each year divided by the mean weekly number of deaths for the same period reported by the end of February in the following year.

Supplemental table 3. Annual population size across different DSP areas during 2015-19

Year	Wuhan DSP (n=3)			Hubei without Wuhan (n=19)			China without Hubei (n=583)		
	Men	Women	Total	Men	Women	Total	Men	Women	Total
**2015**	1108341	1094322	2202663	5265062	5083899	10348961	163552667	156203059	319755726
**2016**	1148420	1131780	2280200	5200237	5015963	10216200	164335157	157124086	321459243
**2017**	1164908	1149361	2314269	5214936	5037277	10252213	165202090	158449944	323652034
**2018**	1150377	1143048	2293425	5158014	5021284	10179298	165653657	159068552	324722209
**2019**	1195157	1105730	2300887	5049926	5106690	10156616	166277297	159610706	325888003

Supplemental Table 4. Observed mortality rates (per 100,000) from selected major diseases in 2020 versus mean of 2015-2019 across different DSP areas

	Wuhan DSP			Hubei without Wuhan			China without Hubei		
**Causes of death**	**2020**	**2015-19**	**Rate ratio**	**2020**	**2015-19**	**Rate ratio**	**2020**	**2015-19**	**Rate ratio**
**All cause**	1147.2	667.8	1.72	867.3	813.8	1.07	675.4	692.6	0.98
**(i) Infectious diseases***	290.1	35.0	8.29	27.6	27.7	1.00	20.3	29.3	0.69
Pneumonia	275.2	20.3	13.58	11.4	13.8	0.82	9.5	16.6	0.57
Unspecified viral	34.6	0.2	141.76	1.8	0.6	2.95	0.4	1.1	0.35
Others pneumonia	48.0	20.0	2.40	5.0	13.2	0.38	9.0	15.5	0.59
COVID-19	192.6	0	–	4.6	0	–	0.05	0	–
Others	14.9	14.7	1.01	16.2	13.9	1.16	10.8	12.7	0.85
**(ii) Non-communicable diseases**	757.6	586.4	1.29	744.1	703.4	1.06	606.7	610.4	0.99
Cancer	186.4	167.0	1.12	179.1	154.1	1.16	153.9	149.0	1.03
CVD	408.1	310.6	1.31	416.1	387.3	1.07	334.0	329.7	1.01
MI	139.9	116.2	1.20	150.4	137.4	1.09	135.9	131.2	1.04
Ischemic stroke	56.6	55.7	1.02	90.8	103.5	0.88	58.5	65.7	0.89
Hemorrhagic stroke	96.9	70.3	1.38	70.0	50.8	1.38	82.2	73.5	1.12
Hypertensive heart disease	59.9	24.8	2.41	48.3	47.2	1.02	24.4	24.7	0.99
Chronic respiratory disease	55.6	46.2	1.20	73.4	103.6	0.71	55.2	72.9	0.76
COPD	45.5	37.6	1.21	64.9	96.8	0.67	50.2	67.7	0.74
Diabetes	45.6	20.2	2.25	17.5	13.1	1.33	18.5	17.2	1.07
CKD	7.2	3.9	1.86	8.5	8.0	1.06	4.3	4.6	0.94
Others	54.6	38.5	1.42	49.5	37.3	1.33	40.8	37.0	1.10
**(iii) Injury**	53.7	37.5	1.43	83.6	77.2	1.08	37.7	42.6	0.88
Traffic accident	4.6	6.6	0.69	9.4	18.4	0.51	8.7	13.4	0.65
Suicide	11.7	6.0	1.94	32.2	23.1	1.40	5.3	5.4	0.98
Fall	24.5	13.0	1.89	22.0	16.1	1.37	12.4	10.7	1.16
Other injuries	13.0	11.9	1.09	20.0	19.6	1.02	11.3	13.1	0.86
**(iv) Other diseases**	45.8	8.9	5.12	11.9	5.5	2.15	10.8	10.4	1.04

* Includes small number of deaths from maternal, perinatal, and nutritional diseases
